# Bioactive Edible Gel Films Based on Wheat Flour and Glucose for Food Packaging Applications

**DOI:** 10.3390/gels10020105

**Published:** 2024-01-27

**Authors:** Argyri-Ioanna Petaloti, Styliani Makri, Dimitris S. Achilias

**Affiliations:** Laboratory of Polymer and Colors Chemistry and Technology, Department of Chemistry, Aristotle University of Thessaloniki, 54124 Thessaloniki, Greece; iriannapetaloti@gmail.com (A.-I.P.); makristyli@chem.auth.gr (S.M.)

**Keywords:** food packaging, edible films, wheat flour, glucose, antioxidant activity

## Abstract

In order to prepare bioactive edible gel films with enhanced properties, the feasibility of using wheat flour as a raw material with glucose added at several concentrations was studied in this investigation. Films were prepared with glucose concentrations of 0.5, 0.7 and 1 g/g of flour and characterized for their physicochemical properties, including water content, solubility, degree of swelling, chemical structure by FT-IR (ATR) spectroscopy, morphology by SEM microscopy, thermal properties by DSC, gas and water vapor permeability and antioxidant activity. Biodegradation studies were also carried out in soil for 27 days and evaluated by weight loss measurements. It was found that the gel film with the higher glucose concentration exhibits a homogeneous and continuous structure with no cracks and no fragility, accompanied by an increased thickness and solubility and a decreased degree of swelling compared to those with lower concentrations. The chemical structure of all films was verified. Moreover, the increase in glucose content leads to better gas barrier properties with lower oxygen, CO_2_ and water vapor transmission rates and increased water vapor permeability. A slightly elevated melting temperature was observed in the films with higher glucose content. Higher antioxidant activity was also associated with higher percentage of glucose. Finally, the biodegradation of the films ranged from 13 to nearly 70%. Therefore, it can be concluded that the addition of glucose to wheat flour in concentration up to 1 g/g could result in edible gel films with excellent properties to be used in food packaging applications.

## 1. Introduction

The development of biodegradable materials for packaging has garnered significant attention in light of growing environmental apprehensions surrounding petroleum-based plastics, which are non-biodegradable and contribute to land and sea pollution. The utilization of synthetic polymers, including poly(lactic acid) and poly(butylene succinate), has been the subject of extensive research and practical application in the realm of food packaging, as are microbial polymers such as polyhydroxyalkanoates and poly β-hydroxybutyrate, produced by natural or genetically modified microorganisms [1,2,3,4]. Polysaccharides, including starches, pectins, alginates, cellulose derivatives, carrageenans, chitosan, gums, fibers, and proteins such as soy proteins, wheat gluten, corn zein, sunflower proteins, whey, gelatin, casein and keratin, are among the biopolymers employed in the formation of edible and/or biodegradable films [5]. Among them, protein molecules have unique functional properties, though their mechanical and barrier properties are inferior to that of plastic-based packaging. Recently, various strategies have been developed for improving the overall performance of biopolymeric food packaging films. Among them is the use of cross-linking agents (such as transglutaminase) in functionalized protein-based food packaging films [1].

Edible gel coatings or gel films, in contrast to conventional food packaging materials, are characterized as thin layers that are applied or enveloped onto the surface of food products [6]. These films or coatings, formulated with one or a combination of these components, can serve as a barrier against mass transfer, thereby prolonging the shelf life of food products [7]. An ideal edible film should possess low water, gas and oil permeabilities and adequate mechanical properties to ensure its integrity is maintained throughout its shelf life [8].

Wheat flour is a highly consumed processed cereal product on a global scale. Its primary macromolecular constituents are starch and gluten, which significantly influence the essential functional and structural attributes of wheat flour [9]. Wheat flour consists mainly of starch, comprising approximately 70–75% of its composition, and protein, which account for approximately 8–14% [10]. In addition to these major components, wheat flour also contains minor constituents such as lipids, which make up approximately 2% of its composition, non-starch polysaccharides, which account for approximately 2–3%, as well as minerals, vitamins, antioxidants and other essential nutrients that are present in whole wheat flour [11]. Starch, as the main component of wheat flour, is composed predominantly of amylose (20–30%) and amylopectin (70–80%) [12,13]. The semicrystalline structure of wheat starch is characterized by a crystalline layer that arises from the graded arrangement of the amylopectin double helix within the starch granules [14]. The physicochemical characteristics of starch undergo hydrogen bond relaxation through the promotion of interaction between starches and the enhancement of crystallization during heating. These properties are also influenced by the levels of moisture and amylose content, as reported in previous studies [15]. Furthermore, amylopectin has been found to facilitate the absorption of water, swelling and gelatinization of starch granules [16].

As the main component of wheat flour, starch is also one of the primary materials used in producing edible films [17]. Nevertheless, starch-based films exhibit certain drawbacks, including low water resistance and low water vapor barriers, which ultimately impact their overall stability and mechanical capabilities [18]. Producing materials with high strength and low water sensitivity can be achieved by incorporating a second biopolymer into the starch-based composite [19]. By adding maize and rice starches, as well as various plasticizers and fillers, the characteristics of bioplastics can be greatly improved [20,21]. Most commonly used plasticizers that can be incorporated are glycerol, sorbitol, diethanolamine and triethanolamine [22]. Films fabricated by using polyethylene glycol as a plasticizer and carrageenan films with various concentrations of arrowroot starch exhibited diverse characteristics at different concentrations [23]. Adding orange peel and glycerol as a plasticizer led to better strength, flexibility and biodegradability [18]. The inclusion of nano-clay in starch-based films resulted in a reduction in water absorption [24]. Furthermore, the integration of antioxidants and/or nanofillers has been implemented in films in order to enhance their antioxidant, flavonoid and montmorillonite nano-clay constituents [5,25,26]. As the flour is composed of natural blends of starch, protein, lipids and fibers, it can be utilized in its unaltered state as a composite for the production of edible films or coatings. The characteristics of a flour film are contingent upon a multitude of natural and inherent molecular interactions that occur between the constituent components of starch, protein, lipid and fiber, as well as the prevailing conditions during the drying and production processes [27,28,29,30]. An additional benefit of incorporating flours in the process of film development is their diverse botanical origins, which consequently results in varying compositions [31].

In this study, the feasibility of using wheat flour together with glucose added at several concentrations for the development of edible gel films was evaluated. The produced gel films were studied in terms of thickness, moisture content, water solubility, degree of swelling, gas and water vapor permeability as well as their optical, thermal and antioxidant properties. The aim was to examine if the addition of glucose would affect the gel film properties. It was found that several properties of the films prepared including antioxidant activity, oxygen and CO_2_ transmission rate and water vapor permeability were enhanced with the addition of glucose. Therefore, it could be concluded that the addition of glucose to wheat flour can result in edible gel films with excellent properties to be used in bioactive food packaging applications.

## 2. Results and Discussion

### 2.1. Physical Properties

As it is reported in the experimental section, three gel films were prepared using wheat flour under three different glucose concentrations, namely 0.5, 0.7 and 1 g/g of flour, and were given the code names Film_A, Film_B and Film_C, respectively. The basic gel film properties, including thickness, water content, solubility and swelling degree, are shown in Table 1. The thickness of the gel films increased alongside an increased amount of glucose added into the solutions, though the values for Film_B and Film_C were not statistically different. Water content (WC) also showed an increase, as it was up to 60% for Film_C compared to Film_A. WC of bio-based film is generally linked to polymer polarity, type of plasticizer [32], process condition and structure of films [33]. The WC of films varied in the range of 7.98–12.91% by raising plasticizer content, while in wheat flour films with glycerol and sorbitol as plasticizers the range was larger (6.19–23.55%) [34]. 

The properties of water solubility and swelling are of significant importance in biodegradable gel films, as they have the potential to affect the water resistance, particularly in environments with high levels of humidity [35]. As shown in Table 1, Film_A and Film_B exhibited a similar solubility and degree of swelling in water at 25 °C after 24 h of immersion. As for Film_C, an increase up to 42.1% of solubility and a decrease up to 30.9% of swelling degree were observed compared to Film_A. For both parameters (solubility and swelling degree) the values of Film_C are statistically different compared to those of Film_A. In general, a plasticized film with a higher content of glycerol exhibited a greater affinity to water due to the hydrophilic properties of the plasticizer [31]. Our obtained films were more soluble than banana flour ones [33].

### 2.2. Chemical Characterization

**Figure 1:**
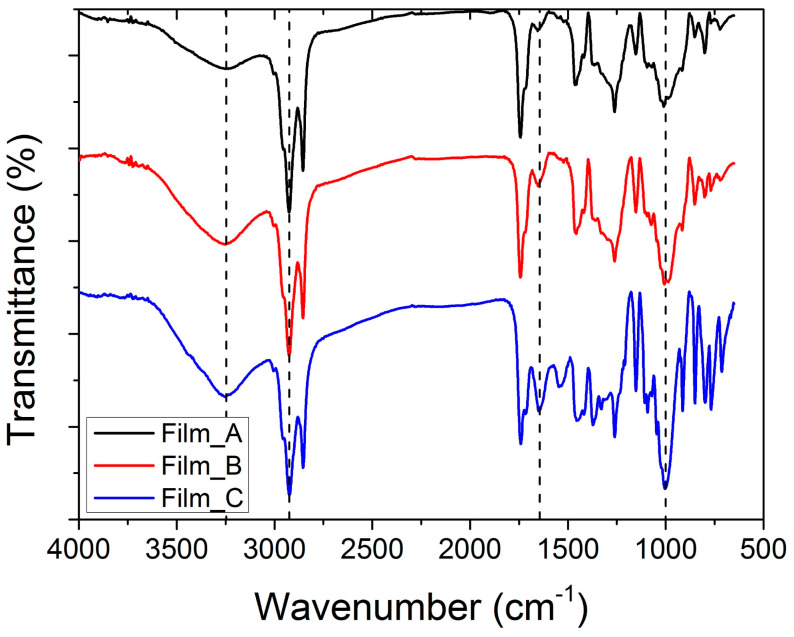
FTIR spectra of films prepared with different glucose content.

The chemical structure of the films prepared was identified through recording of their IR spectra. Figure 1 shows the FTIR spectra for the three films of flour with different glucose contents. Each of the films displayed a distinct peak at approximately 1000 cm^−1^, which corresponds to the characteristic absorption peak of polysaccharide molecules [36]. This peak is attributed to the C–O and C–C tensile vibrations. The absorption peaks observed at 1550 cm^−1^ and 1650 cm^−1^ are indicative of the C=O and C=N functional groups present in proteins, respectively [37,38]. The spectral band observed within the range of 2800–3000 cm^−1^ is commonly associated with the stretching of the –C–H (CH_2_) group present in the protein [37]. The spectral band that is situated within the range of 3100–3700 cm^−1^ is commonly associated with the fundamental stretching mode of the –OH group. This particular band is derived from the polysaccharide molecules as well as certain amino acids found in protein [39]. In general, the occurrence of hydrogen bonding results in a displacement of the characteristic peak towards a lower wavenumber position [40]. As observed, the band located in the ranges of 3100–3700 cm^−1^ increases with increasing glucose addition. This band is less intense for film_A and more characteristic for film_C.

### 2.3. Morphological Observations

**Figure 2:**
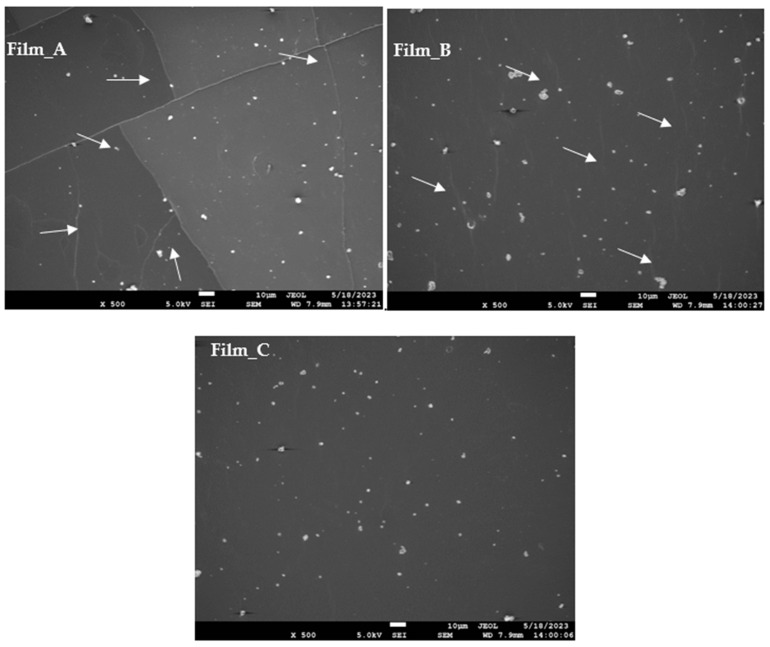
SEM photographs of films with different amounts of glycose.

Figure 2 presents SEM photos of all wheat flour gel films. All the films are homogeneous in their morphology. The present study reveals that Film_A, characterized by a lower glucose concentration, exhibits a non-continuous structure with many fissures, thereby indicating its inherent fragility. Conversely, Film_B displays a more continuous structure with only a few cracks. Notably, Film_C, which has a higher glucose concentration, exhibits a superior structure with no cracks and no fragility. In analogous investigations, the utilization of glycerol as a plasticizing agent at elevated concentrations yielded commensurate outcomes, as a homogeneous and continuous structure is seen with no pores or cracks [31]. A similar observation in wheat flour films with glycerol and sorbitol as plasticizers led to similar results. Μore specifically, un-plasticized films exhibited a non-uniform surface with loose structural integrity. By contrast, the incorporation of glycerol and sorbitol resulted in a more uniform surface [34].

### 2.4. Gas and Vapor Permeability

The preservation of food quality relies on the transfer of water vapor through packaging material. Therefore, there is a high demand for bio-based films with low water permeability. The gas permeability measurement was not possible for Film_A due to its extreme brittleness. The study was carried out for the other two films, which did not have a brittleness problem. Permeability to oxygen and carbon dioxide was studied and the results are presented in Table 2. As observed, the increase in glucose content led to lower OΤR and CO_2_TR and better gas barrier properties. In both parameters, the values measured for Film_C are statistically different compared to those for Film_B. Wheat gluten-based gel coatings and films exhibit good oxygen barrier characteristics owing to their elevated resistance to nonpolar compounds, including O_2_, CO_2_ and lipids [41].

**Figure 3:**
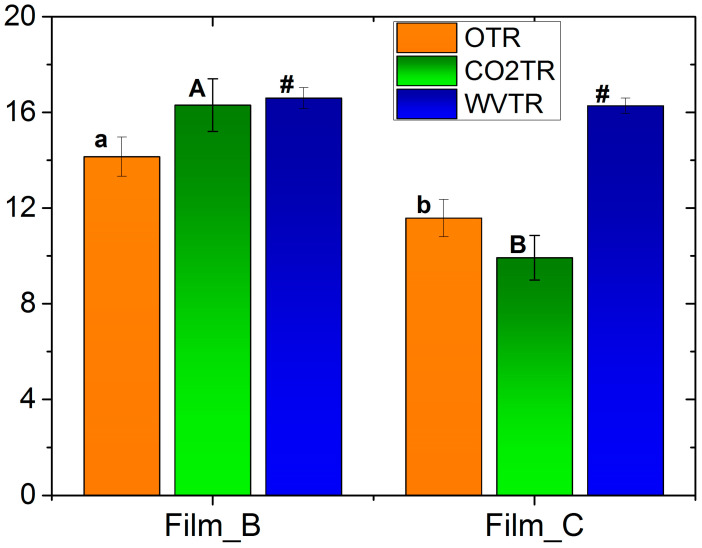
Comparative measurements of oxygen, CO_2_ and water vapor transmission rate for Film_B and Film_C. Different letters or symbols indicate statistically significant difference (*p* < 0.05).

Regarding the water vapor permeability of flour gel films, measurements are presented in Table 3. Due to the fragility of Film_A, measurement for this film was not possible. Assisted by the methodology, water vapor transmission rate (WVTR), water vapor permeability (WVP) and percentage of water that penetrates were calculated for Film_B and Film_C. The increase in glucose to the film led to a decrease of the WVTR up to 1.9% and an increase for water vapor permeability and percentage of water that penetrates up to 19.9% (as expected, as glucose by its nature tends to absorb water vapor from the atmosphere) and 3.9%, respectively. The values of WVTR were not statistically different between Film_B and Film_C whereas they were different concerning the water vapor permeability. The obtained values of WVP in the present work were lower than those with glycerol and sorbitol as plasticizers and for banana- [33] and rice flour-based films [29]. The impact of glycerol incorporation into wheat gluten films has examined and revealed that the inclusion of glycerol as a plasticizer resulted in an elevation of the films’ water vapor permeability [42]. Figure 3, shows comparative measurements of OTR, CO_2_TR and WVTR for Film_B and Film_C, respectively. 

### 2.5. Thermal Behaviour

**Figure 4:**
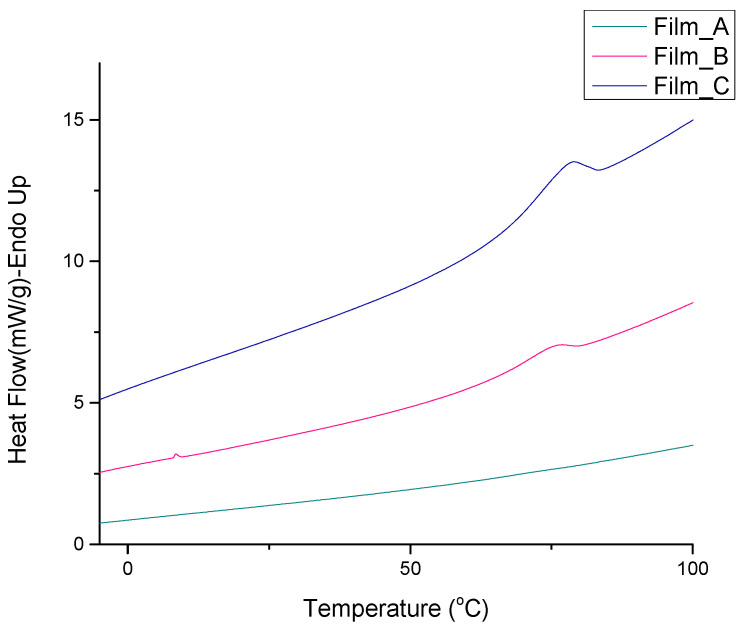
DSC scans obtained during heating of Film_A, Film_B and Film_C.

The thermal behavior of wheat flour gel films was investigated by differential scanning calorimetry (DSC). From the heat scans shown in Figure 4, the glass transition and cold crystallization temperatures were not detected. Endothermic phenomena were observed for Film_B and Film_C corresponding to melting temperatures. Film_A did not exhibit a discernible melting point. In contrast, Film_B displayed a melting peak at 75.33 °C, accompanied by a ΔH value of 2.14 J/g. Notably, Film_C, which has a higher glucose concentration, exhibited an elevated melting point of 78 °C and a ΔH value of 9.8 J/g. The experimental results indicate that the presence of glucose resulted in a higher melting point.

### 2.6. Antioxidant Activity

**Figure 5:**
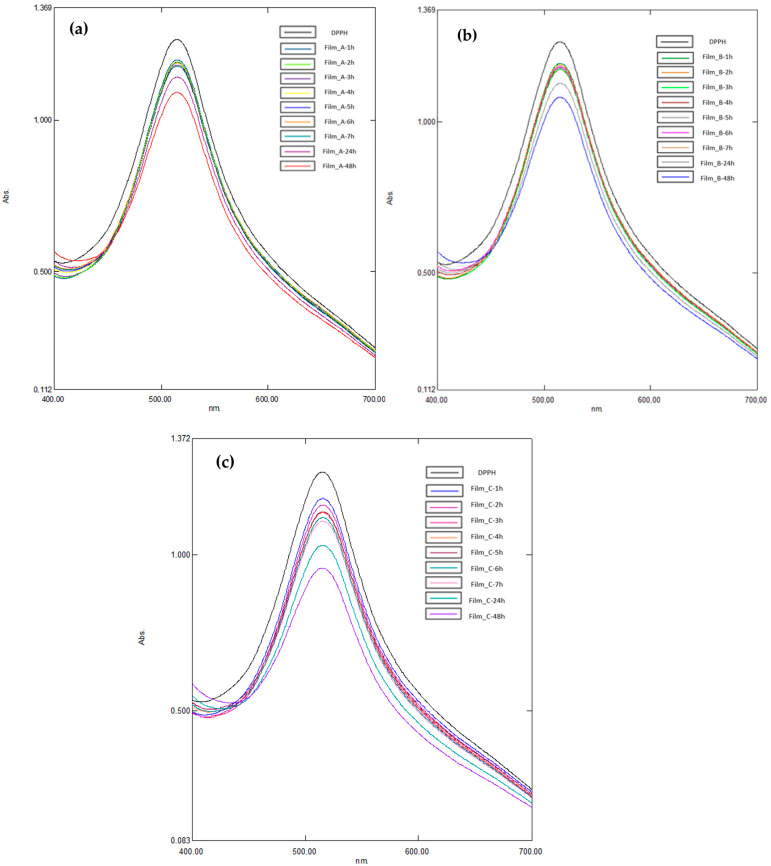
UV spectra obtained from Films_A (**a**), B (**b**) and C (**c**) obtained during antioxidant stability measurements with the DPPH method at several time steps.

The evaluation of the antioxidant stability of the materials prepared was carried out through the utilization of the DPPH method, a widely employed technique for predicting the antioxidant activity of packaging films [43]. The initial color of the samples was observed to be purple, with a transition to yellowish indicating the presence of antioxidant activity attributed to the additive. To quantify the color change, the UV absorbance of the samples was measured and the UV spectra of the materials under investigation are presented in Figure 5.

**Figure 6:**
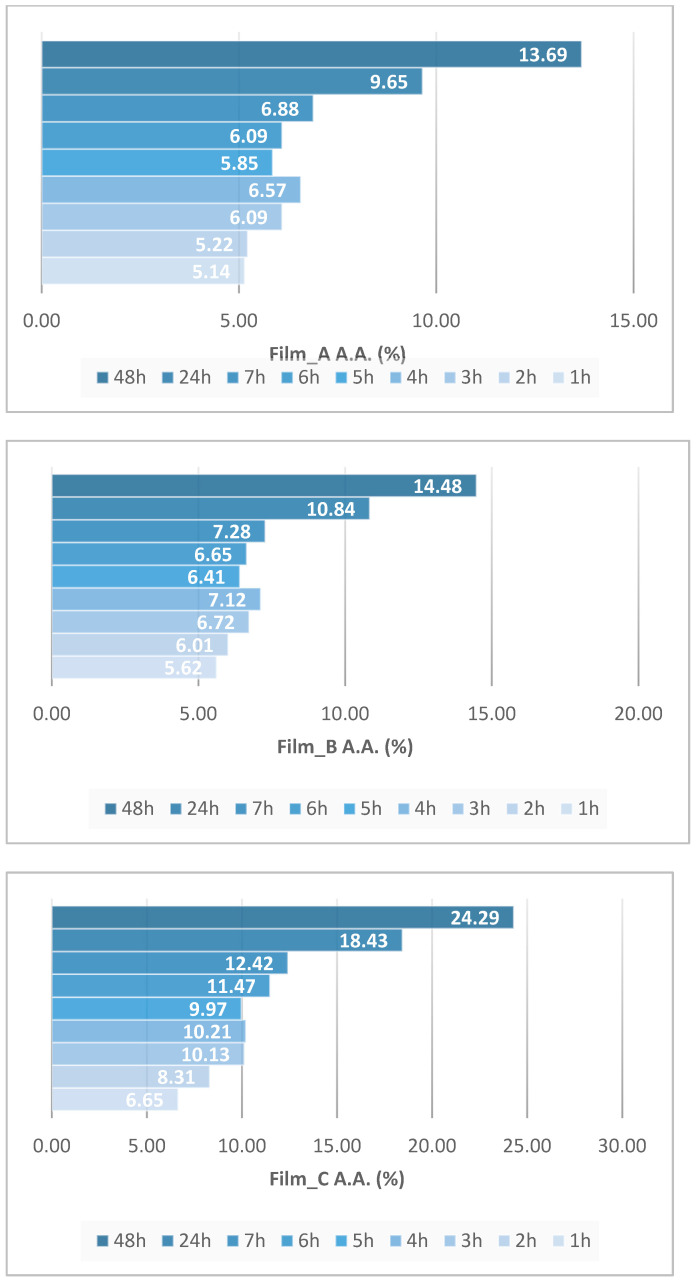
Antioxidant activity (A.A.) of Film_A, Film_B and Film_C at different time steps.

**Figure 7:**
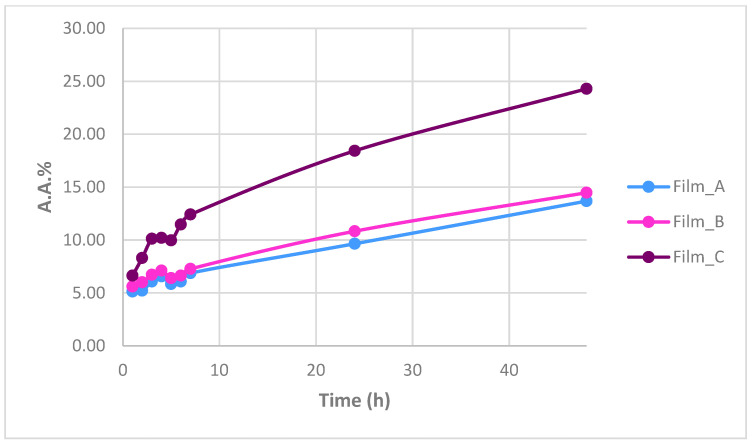
Variation of antioxidant capacity over time for the three films.

Figure 6 shows the percentage of antioxidant activity (%) for every film separately over time. Antioxidant activity is increased when time goes by and is associated with the increasing percentage of glucose. The flour is derived from wheat, which is an important source of bioactive compounds and its incorporation into films seems to lead to antioxidant properties. Its high protein content compared to other cereals seems to also enhance the antioxidant properties of the films [31]. In Figure 7, there is a diagram about the change in antioxidant capacity over time for the three films. A higher antioxidant ability of 24.29% was observed in Film_C, which contains the higher percentage of glucose. 

### 2.7. Biodegradation Study

The significance of soil in the process of biodegradation plays an important role. The biodegradation of films is contingent upon several critical parameters, including temperature, humidity, chemical composition, pH, geographical location and the microbiological activity of the soil [44]. Consequently, the soil employed for the biodegradation investigation was characterized based on its moisture content and pH levels. The soil percentage of humidity, calculated by Equation (8), was 24.86% and the pH was 6.5. The percentage of humidity in soil is subject to the influence of various factors including weather conditions such as temperature and rainfall, irrigation practices and the composition and particle size distribution of the soil. These factors collectively determine the soil’s water-holding capacity, which in turn affects the soil’s moisture content [44].

**Figure 8:**
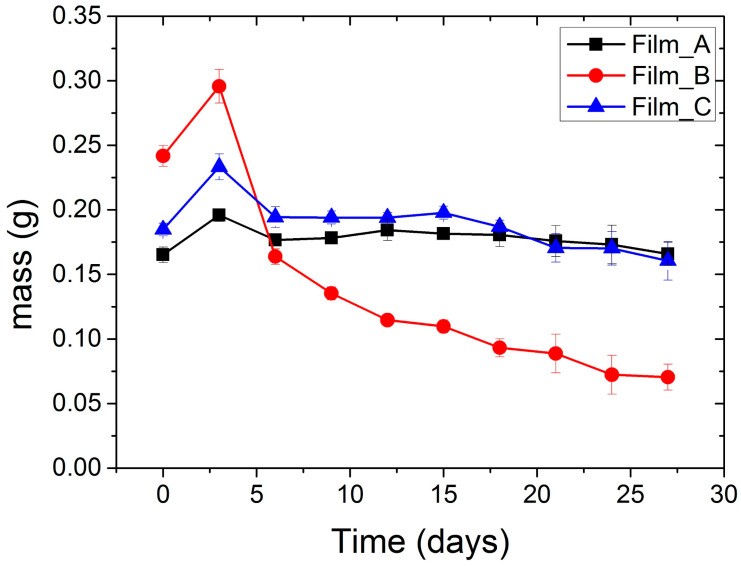
Variation of the mass of each film (A, B and C) with time during their biodegradation in soil.

Biodegradation study of flour films was carried out in soil for 27 days. The biodegradation rate of films was evaluated by weight loss measurements. Table 4 summarizes the weight loss of films during the period of biodegradation. These data are also illustrated in Figure 8. 

As observed, after the first three days, the weight of the films increased due to the absorption of soil moisture. Soil adhered to the surface of the films and its complete removal was difficult. Film_A has the same weight from the first day. Film_C has a decrease to its weight over the days. Film_B, although it did not have the largest amount of glucose, led to a greater reduction in its mass. This is also due to the breaking of the sample. The percentage of weight loss of Film_C at the time of 27 days was 13.10% and Film_B had a decrease of up to 70.87%.

Finally, Table 5 shows the photographs of Film_A, Film_B and Film_C before and after each period of the biodegradation study. Higher decomposition was observed for Film_B and was not associated with the increase of glucose levels. In relation to the macroscopic analysis of samples after 27 days of biodegradation, the physical integrity of films were not preserved. Moreover, all films were more brittle (less flexible) during biodegradation. Weight loss measurements confirmed visual analysis results.

## 3. Conclusions

In order to enhance the properties of edible gel films formed by wheat flour, the addition of glucose at several concentrations was studied in this investigation. It was found that all film properties were affected by glucose concentration. More specifically, thickness, moisture content and solubility were increased with the increasing of the glucose content, in contrast to swelling degree. As observed, the augmentation of glucose concentration resulted in better gas and water vapor permeability, a more homogenous structure and higher antioxidant activity. All films presented a reduction in their mass during the biodegradation study in soil. From all the experimental measurements it was clear that the addition of glucose to flour in concentrations up to 1 g/g could result in edible films with excellent properties to be used in bioactive food packaging applications. In the future, flours of different botanical origin and the addition of various additives could be considered for the incorporation of flours in the film development process.

## 4. Materials and Methods

### 4.1. Materials

The wheat flour was procured by a local company “Afoi Keramari MANNA” (Thessaloniki, Greece). According to the company, the raw material of flour is from Western Macedonian grain (Kozani, Grevena). The composition of wheat flour per 100 g included: fat 1.5 g (of which saturated 0 g), carbohydrates 75 g (of which sugars 0 g), edible fibers 0.6 g, proteins 12 g, salt 0.002 g). The glucose that was used was from Sigma Aldrich. Distilled water was used throughout the experiments. 

### 4.2. Preparation of Films

**Figure 9:**
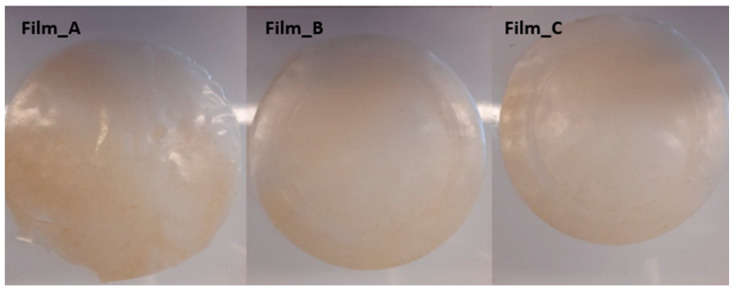
Gel films prepared from wheat flour and different concentrations of glucose. The code names Film_A, Film_B and Film_C were given for glucose concentrations 0.5, 0.7 and 1 g/g of flour, respectively.

Gel films were prepared using flour under three different glucose concentrations (0.5, 0.7 and 1 g/g of flour) (Figure 9). The experimental procedure was used from Drakos et al. [31] with modifications. The concentration of flour, for all the films, was 6 *w*/*v* (dry weight). The required amount of flour and glucose was added to 120 mL of distilled water. The mixture was stirred for 10 min to facilitate the dissolution of the flour. Subsequently, it was subjected to a temperature of 90 °C and maintained at that level for a period of 10 min. For film preparation, the solution was poured onto a silicone mold (27 cm diameter) and dried in a vacuum oven at 40 °C for 24 h. Three films were studied per formulation. Films were put in a desiccator with silica gel until analysis.

### 4.3. Film Characterization

#### 4.3.1. Thickness

A handheld micrometer (with an accuracy of 0.01 mm) was used to measure the film thickness. Six replicates were performed at random positions for each sample treatment. 

#### 4.3.2. Water Content (WC)

In order to measure the WC of the gel films, a 1 cm × 1 cm piece of the film was cut off and its weight was measured before and after being placed in the oven (105 °C for 24 h) [45]. To determine the water content (%), three replicate measurements were made for each film using the following equation:

(1) $$Water\;content\left(\%\right)=\frac{M_{o}-M}{M_{o}}\times 100$$

where *M_o_* was the initial mass (g) and *M* was the bone-dry mass (g).

#### 4.3.3. Solubility and Swelling Degree

The solubility and swelling properties of the gel films were evaluated using the methodologies described by Silva et al. [46] and Zhong et al. [47], with certain modifications [32]. Film samples measuring 1 cm × 1 cm were subjected to a drying process at 70 °C for 24 h in a vacuum oven to obtain the initial dry mass (*M*_1_). Subsequently, the films were placed in 50 mL beakers containing 30 mL of distilled water, covered with plastic wraps and stored at 25 °C for 24 h. After the allotted time, any remaining water in the beakers was discarded and the residual film samples were superficially dried using filter paper. The residual film samples (*M*_2_) were then subjected to another round of drying at 70 °C for 24 h in a vacuum oven to determine the final dry mass (*M*_3_). Three measurements were taken for each film sample for accuracy and consistency. Film solubility and swelling degree were calculated by using the following equations, respectively:

(2) $$Film\;Solubility=\frac{M_{1}-M_{3}}{M_{1}}$$

(3) $$Swelling\;degree=\frac{M_{2}-M_{1}}{M_{1}}$$

#### 4.3.4. FT-IR (ATR)

The Spectrum 1 spectrophotometer from Perkin Elmer (Perkin Elmer, Shelton, CT, USA), equipped with an attenuated total reflectance (ATR) device, was employed as the analytical instrument. Thin films were utilized for the measurements and spectra were recorded over the range of 4000–650 cm^−1^ at a resolution of 2 cm^−1^. To minimize noise, 32 scans were averaged. The instrument’s software (Spectrum v5.0.1) was utilized to identify multiple peaks.

#### 4.3.5. Surface Morphology (SEM)

The scanning electron microscope is a FESEM JSM 7610 FPlus (JEOL, Tokyo, Japan) type. The instrument resolution is 5 nm. The analysis of the samples requires them to be conductive. For this purpose, the samples are carbonized in a carbon sublimation device.

#### 4.3.6. Gas Permeability

The oxygen permeability of the prepared films was evaluated using gas permeability analyzer model: N500 instrument (Guangzhou Biaoji Packaging Equipment Co., Guangzhou, China) in the following conditions: constant temperature 23 °C, relative humidity 0% and gas flow 10 mL/min.

#### 4.3.7. Water Vapor Permeability

The water vapor permeability was assessed in accordance with the ASTM E96 standard [48]. The present experimental configuration employed glass petri dishes measuring 6 cm in diameter and 3 cm in height. Each dish was filled with 10 mL of distilled water, thereby creating a humidity level of 100% within the dish that surpassed the ambient humidity. Petri dishes were then placed within a desiccator containing active silica gel. Prior to being placed in the desiccator, the initial weight of each dish was measured and recorded. Subsequently, the dishes were subjected to a 24 h period within the desiccator, during which their weights were periodically measured. The thickness of each film was accurately measured at six random points and utilized for the ensuing calculations. The mass loss of the cup was determined to be equivalent to the amount of water that had passed through the film. To calculate the permeability to water vapor, the curve of the water passing through the film was initially plotted over a time unit and the slope of the curve was calculated within the linear portion of the curve (Δ*m*/Δ*t*).

*Slope of the water passing through the film curve* = Δ*m*/Δ*t* (4)

The slope of the curve within the linear portion is representative of the water vapor transmission rate WVTR). The resulting value was then divided by the area of the film, *A*, yielding the WVTR (Equation (5)).

WVTR = (Δ*m*/Δ*t*)/*A* = (*Slope of the water passing through the film curve*)/(*area of film*) (5)

In order to obtain the water vapor permeability (WVP) according to Equation (6), the WVTR value is multiplied by the thickness of the film (*d_film_*). The resulting value is divided by the pressure difference in the water vapor on the two sides of the film (Τ = 20 °C, Δ*p* = 2339 Pa).

(6) $$WVP=\frac{WVTR\times d_{film}}{\Delta p}=\frac{\left[\left(\Delta m/\Delta t\right)/A\right]\times d_{film}}{\Delta p}$$

#### 4.3.8. Differential Scanning Calorimetry (DSC)

In order to estimate the melting temperature of every material prepared, the DSC-Diamond (Perkin–Elmer) was used. Approximately 5–6 mg of each sample were weighed, put into the standard Perkin–Elmer sample pan, sealed and placed into the appropriate position of the instrument. Subsequently, they were initially heated from −25 °C to 200 °C at a rate of 10 °C min^−1^. Following this, the samples were cooled to −25 °C at a rate of 10 °C min^−1^.

#### 4.3.9. Antioxidant Activity

The present study assessed the antioxidant activity of the films using the DPPH (2,2-diphenyl-1-picrylhydrazyl) test. To this end, 6 mg of each film was introduced into a glass vial containing 3 mL of DPPH solution and incubated at 25 °C for 24 h in the absence of light. The reaction kinetics were monitored by measuring the disappearance of the DPPH• reactant through absorbance measurements ranging from 400 to 700 nm, with a maximum absorbance at 516 nm, using a Shimadzu Spectrophotometer UV-1800. The percentage of antioxidant activity (*AA*) of the films was determined by measuring the absorbance (*ABS*) at 516 nm, with the absorbance of the DPPH solution serving as the control according to the following equation:

(7) $$AA\left(\%\right)=\frac{{ABS}_{control}-{ABS}_{sample}}{{ABS}_{control}}\times 100$$

#### 4.3.10. Aerobic Biodegradation

Samples were cut into 2.5 cm^2^ pieces to obtain a uniform sample size for degradation. The samples were weighed to record their initial weight and buried in the soil at a depth of about 5 cm. The test was carried out at environment temperature and water was sprinkled on the soil surface every three days to ensure that the soil remained humid. 

The percentage of soil humidity (*h*) was calculated by the weight difference before (*m*_1_) and after (*m*_2_) drying the soil at 105 °C for 24 h, divided by the dried soil mass (Equation (8)). The average of triplicates was used to determine the percentage of humidity [49].

(8) $$h=\frac{m_{1}-m_{2}}{m_{1}}\times 100$$

where:

*h*: Percentage of humidity

*m*_1_: Mass of wet soil

*m*_2_: Mass of dried soil

In addition, the pH of soil was measured.

The samples were measured for weight loss every 3 days from the day they were initially buried. The following equation was used to determine the percentage of weight loss:

(9) $$Weight\;loss=\frac{M_{i}-M_{f}}{M_{i}}\times 100$$

where:

*M_i_*: mass at day 0

*M_f_*: mass at day *Y*

*Y*: is the period after biodegradation (3, 6, 9, 12, 15, 18, 21, 24, 27)

#### 4.3.11. Statistical Analysis

All data were analyzed in triplicates. One-way analysis of variance (ANOVA) was applied to determine statistical significance between the three films studied. Statistical analysis was performed using IBM SPSS Statistics 28. The statistical significance level was set at *p*-value ≤ 0.05.

## Figures and Tables

**Table 1:** Physical properties (mean and standard deviation) of the gel films prepared. Different superscripts indicate statistically significant difference (*p* < 0.05).

	Thickness (μm)	Water Content (%)	Solubility	Swelling Degree
Film_A	146.7 ± 11 ^a^	7.98 ± 1.74 ^a^	0.38 ± 0.15 ^a^	2.33 ± 0.21 ^a^
Film_Β	180.8 ± 14 ^b^	12.77 ± 0.87 ^b^	0.40 ± 0.12 ^a^	2.07 ± 0.18 ^a^
Film_C	182.5 ± 7 ^b^	12.91 ± 0.94 ^b^	0.54 ± 0.16 ^b^	1.61 ± 0.15 ^b^

**Table 2:** Oxygen and CO_2_ permeability measurements of Film_B and Film_C. Different superscripts indicate statistically significant difference (*p* < 0.05).

	Oxygen Permeability, OTR [cm³/(m^2^ × days × 0.1 MPa)]	Carbon Dioxide Permeability, CO_2_TR [cm³/(m^2^ × days × 0.1 MPa)]
Film_B	14.147 ± 0.82 ^a^	16.302 ± 1.10 ^a^
Film_C	11.583 ± 0.78 ^b^	9.925 ± 0.93 ^b^

**Table 3:** Water vapor transmission rate and permeability of Film_B and Film_C. Different superscripts indicate statistically significant difference (*p* < 0.05).

	Water Vapor Transmission Rate WVTR, (g/(m^2^ × h)	Water Vapor Permeability WVP. (g/m × h × bar)	Percentage of Water That Penetrates (24 h) (%)
Film_B	16.596 ± 0.44 ^a^	1040×10^−6^ ± 0.08 ^a^	2190 ± 0.23 ^a^
Film_C	16.277 ± 0.31 ^a^	1247×10^−6^ ± 0.15 ^b^	2275 ± 0.17 ^a^

**Table 4:** Variation of the mass of each film (A, B and C) with time during their biodegradation in soil.

Time (Days)	Film_A	Film_B	Film_C
0	0.1652 ± 0.006	0.2417 ± 0.008	0.1847 ± 0.005
3	0.1959 ± 0.005	0.2956 ± 0.013	0.2332 ± 0.010
6	0.1767 ± 0.003	0.1638 ± 0.006	0.1943 ± 0.008
9	0.1781 ± 0.003	0.1353 ± 0.005	0.1940 ± 0.005
12	0.1843 ± 0.008	0.1146 ± 0.004	0.1939 ± 0.004
15	0.1816 ± 0.004	0.1097 ± 0.005	0.1977 ± 0.005
18	0.1805 ± 0.009	0.0932 ± 0.007	0.1869 ± 0.005
21	0.1759 ± 0.012	0.0887 ± 0.015	0.1705 ± 0.011
24	0.1731 ± 0.015	0.0723 ± 0.015	0.1702 ± 0.013
27	0.1659 ± 0.009	0.0704 ± 0.010	0.1605 ± 0.015

**Table 5:** Photographs of Film_A, Film_B and Film_C at different time periods of their biodegradation.

Time (Days)	Film_A	Film_B	Film_C
3	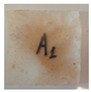	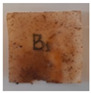	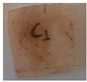
6	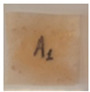	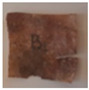	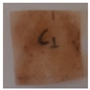
9	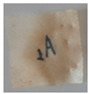	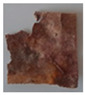	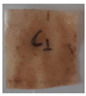
12	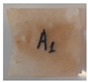	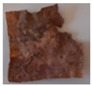	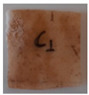
15	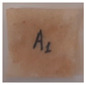	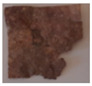	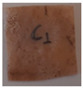
18	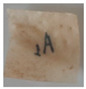	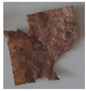	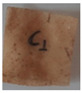
21	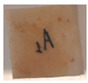	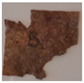	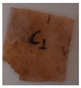
24	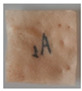	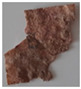	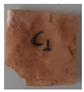
27	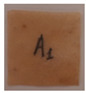	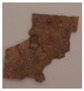	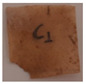

## Data Availability

All data and materials are available upon request from the corresponding author. The data are not publicly available due to ongoing research using the data.
